# Acceptance and use of virtual reality games: an extension of HMSAM

**DOI:** 10.1007/s10055-023-00749-4

**Published:** 2023-01-31

**Authors:** Tuomas Kari, Mehmet Kosa

**Affiliations:** 1 Natural Resources Institute Finland (Luke), Helsinki, Finland; 2 Institute for Advanced Management Systems Research, Turku, Finland; 3 Department of Psychology, Arizona State University, Tempe, AZ USA; 4 College of Arts, Media and Design, Northeastern University, Boston, MA USA

**Keywords:** Virtual reality, Digital games, User behavior, Virtual reality gaming, Dual-purposed systems, VR

## Abstract

Virtual reality (VR) is considered as one of the technological megatrends of 2020s, and today, VR systems are used in various settings, digital gaming being among the most popular ones. However, there has been a dearth of understanding regarding the central factors behind VR gaming acceptance and use. The present study therefore aimed to explain the factors that drive the use and acceptance of VR games. We extended the hedonic-motivation system acceptance model with utilitarian and inconvenience factors to capture the pertinent features of VR systems more holistically. We proposed a theoretical model and analyzed it through covariance-based structural equation modeling using an online survey sample of 473 VR gamers. Our findings help explain the role of different antecedents behind VR gaming acceptance and demonstrate that VR gaming is driven more by the hedonic gaming aspects than by the utilitarian health and well-being aspects of VR games, enjoyment being the strongest driver behind VR gaming intention and immersion. Moreover, findings also suggested that use intentions and immersion levels are not significantly diminished by physical discomfort and VR sickness. The findings, which potentially extend to other VR systems as well, also pose important implications for the providers of VR games. As the main contribution, based on our empirical findings, we provide a greater theoretical understanding on VR gaming acceptance and use.

## Introduction

Advancement in information technology has had a significant impact on digital gaming and entertainment. Novel sensors and other technological innovations have facilitated the design and development of new digital gaming concepts, which provide a wide variety of interaction possibilities to their users. One such digital gaming concept that has benefited greatly from the recent technological advancements is virtual reality (VR) gaming. Burdea and Coiffet (1994), in their classic book *Virtual Reality Technology,* defined VR as “a high-end user-computer interface that involves real-time simulation and interactions through multiple sensorial channels.” In general, VR systems are designed to produce realistic virtual environments. This is commonly done by utilizing image, sound, haptic, and other sensations. Typical aim is to provide an experience that immerses the user (Flavián et al. 2019; Sherman and Craig 2018), that is, “an experience of total engagement where other attentional demands are, in essence, ignored” (Agarwal and Karahanna 2000). VR is considered as one of the technological megatrends of 2020s (Xi and Hamari 2021), and today, VR systems are used in various settings, digital gaming being one of them.

During the recent decade, different types of VR games have emerged. The most typical ones are those that are played wearing a headset. The most common gaming platforms include game consoles, computers, and smartphones connected to VR headsets, standalone VR systems, as well as VR arcades (Grand View Research 2020a). As VR technology and games have become more accessible to general public, their popularity has increased steadily. While the market increase has been slower than anticipated few years back (Statista 2016, 2021), the expectations for future growth are high: The global VR gaming market size was valued at USD 11.56 billion in 2019 and is anticipated to grow at a compound annual growth rate of 30.2% from 2020 to 2027 (Grand View Research 2020a). Moreover, recent metaverse initiatives (e.g., Facebook, newly renamed as Meta) can further add to VR use becoming more common in the future (Verge 2021) To understand and enable this growth along with mainstream adoption, it is imperative to investigate what factors drive the use and acceptance of VR gaming.

Along with the rising public interest, the academic interest toward investigating VR gaming has also grown. However, there has been a dearth of understanding regarding the central factors behind VR gaming acceptance and use. We are aware of only few studies (Jang and Park 2019; Kosa et al. 2020; Tsai et al. 2021) that have investigated the acceptance of games that utilize VR equipment. While these acceptance studies provide important first insights on VR gaming acceptance and use, they have taken the approach where VR games are seen mainly as hedonic systems and focused on their leisure use. Therefore, there is a general gap in VR gaming research regarding the acceptance of VR games as dual-purposed systems. Further, the inconvenience factors, such as VR sickness and VR discomfort, are mostly overlooked in the former acceptance studies. Moreover, there is a limited understanding of the motives driving VR adoption (Steffen et al. 2019). In the present study, we provide a greater understanding of VR gaming acceptance by investigating VR games as dual-purposed systems (Hong and Tam 2006) affording both hedonic and utilitarian benefits to their users and including inconvenience factors.

As the VR gaming technology evolves, it affords new ways of playing (e.g., new interaction modalities), novel experiences (e.g., deeper immersion and presence), and multifaceted benefits (e.g., utilitarian benefits in addition to hedonic benefits). VR technology also affords new types of activities where the laws and limits of physical reality can be overcome (see Steffen et al. 2019). Thus, there is a prevalent need to study the usage aspects of VR games, with these new affordances in mind. The understanding of these aspects is highly important for the designers, developers, and marketers of VR games for them to provide such VR gaming solutions that people genuinely want to use and invest in. This would subsequently advance the adoption and diffusion of VR gaming systems. Moreover, this adoption trend would most likely also extend toward VR systems in general, as gaming is considered to be a central driving force in the development and adoption of VR technology and systems (Jabil 2018).

The present study addresses the aforementioned research gap concerning the acceptance and use of VR games by investigating what kinds of factors explain the intention to use VR games. The main research question that the study aims to answer is: What are the factors explaining the use intentions of VR games? The focus is on VR games that are played using VR headsets, as those are currently the most common and accessible options for the users. In order to answer this question, we follow the hypothetico-deductive research method, as we first propose a new theoretical model for explaining the usage intentions of VR games and then empirically test this model by analyzing data collected from 473 VR gamers using structural equation modeling (SEM). From a theoretical perspective, our approach considers VR games as dual-purposed systems.

## Related research and background

In this section, we first present the technology used to play VR games. Then, we continue by reviewing previous research on the acceptance of digital games in general and by discussing VR gaming literature to pinpoint the relevant research gap. We also present potential benefits of VR gaming research and demonstrate that there is a lack of knowledge about VR gaming acceptance.

### Virtual reality gaming systems

The common aim of using VR systems is to provide sensory immersive experiences (Flavián et al. 2019) by simulating users' physical presence in a given virtual environment (Sherman and Craig 2018). To provide such experiences, most current VR systems utilize VR headsets that are equipped with numerous sensors and are connected to different technologies (e.g., different platforms and motion sensing systems) and VR accessories (e.g., controllers, bodysuits, and gloves). However, as pointed out by Sherman and Craig (2018), a headset is not the only way to deliver a VR experience. Depending on the definition, VR can include different kinds of systems (Sherman and Craig 2018). For example, VR experience can also be produced by using stereoscopic displays (e.g., Mostafa et al. 2017) or the CAVE automated virtual environments (Cruz-Neira et al. 1993). Nevertheless, using a headset is by far the most common way for providing immersive VR experiences (Sherman and Craig 2018).

A VR headset (a.k.a. head-mounted display) is a type of display device that is worn on the head and where the display is positioned right in front of the user's eyes no matter where the user’s head may turn (Techopedia 2017). Different types of VR headsets vary on their level of technology. A key component is the used tracking system, referring to the process of determining users’ viewpoint position and orientation (Angelov et al. 2020). Tracking systems are generally divided into two types: orientational and positional. The type of tracking determines the number of degrees of freedom (DoF) within which changes in the position of the target devices are tracked, whereas orientational tracking systems determine the orientation of the devices (headset and controllers) in three-dimensional space (3DoF), in positional systems also the spatial position of the devices is determined (6DoF), allowing for more realistic user interactions (Angelov et al. 2020). Obviously, VR systems also vary on the experience they provide. In general, positional tracking is preferable to achieve full-immersion experience in VR (Angelov et al. 2020). Moreover, ergonomics such as the weight of the headset, in addition to the fidelity of the technology, influence the level of immersion and the consequent experience (Angelov et al. 2020; Cummings and Bailenson 2016; Muhanna 2015).

The most common way to play VR games is with a VR system that includes a headset and controllers. Game consoles, computers, and even smartphones and tablets can be connected to VR headsets. The connection can be either tethered or untethered. Tethered headset refers to a headset that is physically connected to the computing device by cables, whereas untethered headset refers to a headset that is either standalone or wirelessly connected to the computing device. Some tethered VR headsets can be transformed to untethered by using wireless adapters (Kim and Yun 2020). Unlike tethered and some untethered headsets, standalone headsets contain all the required computing hardware to produce the VR content (i.e., run the software) without needing to connect to an external computing hardware (Angelov et al. 2020). Both tethered and untethered headsets are common in VR gaming these days, but there seems to be a trend toward standalone headsets (Grand View Research 2020b) since they are usually easier to setup and start, and they make the experience safer for users by freeing them from extra cables or equipment.

In addition to home settings, another popular setting to play VR games is VR arcades (Grand View Research 2020a). Numerous VR arcades have been founded around the globe during the past years and their popularity has been on a consistent rise (Forbes 2018; Venturebeat 2018). There are various types of VR games that different VR arcades provide. For example, there are VR arcades that provide VR headsets and controllers along with a small space for the user to move around in, VR motion platforms, or free-roam VR spaces. Free-roam VR typically utilizes either standalone headsets or headsets connected to computers that the users carry in a backpack while playing, thus allowing them to move freely inside the designated gaming area (Kari 2019). Besides the aforementioned most common types of VR technologies to play VR games, other types of VR technologies also exist. For an extensive overview of VR technologies, definitions, and history, we refer the interested reader to the work of Sherman and Craig (2018).

### Digital gaming acceptance research

Various studies have investigated the acceptance of digital games. For example, Lowry et al. (2013), studying hedonic-motivation systems by using games in their experiments, found that enjoyment and curiosity increase immersion and intention to use, whereas control increases only the immersion, and perceived usefulness increases only the intention to use. They also found that perceived ease of use is best represented as an indirect predictor of intention to use, fully mediated by enjoyment, perceived usefulness, and curiosity.

Hsu and Lu (2004), who studied the acceptance of online games, found that the intentions to play were directly affected by social norms, flow experience,^1^ and attitude. The attitude, on the other hand, was predicted by critical mass, perceived ease of use, and perceived usefulness. Notably, perceived usefulness did not have a direct effect on the intention to play.

Kari and Makkonen (2014), who studied the acceptance of exergames, found that perceived behavioral control, descriptive subjective norm, and attitude are important drivers behind the intention to use exergames. They also measured how the beliefs on the outcomes of use affect the attitude toward intention to use exergames and found that this intention is driven more by the hedonic than the utilitarian aspects of exergaming. On a similar note, Lin et al. (2012) found that both perceived enjoyment and perceived exercise utility influence behavioral intention to use exergames, but the influence of perceived enjoyment is greater than the influence of perceived exercise utility.

Chang et al. (2014) presented a model to explain continuance intention of online multiplayer games integrating cognitive, affective, and social influence factors. They found that continuance intention was predicted by both utilitarian and hedonic outcome expectations with the hedonic outcome expectations having a greater effect of these two. Both of these relationships were moderated by flow experience. They also found that critical mass and subjective norm predicted continuance intention. Similarly, Lowry et al. (2015) found that expectations and confirmation/disconfirmation of both joy and productivity related motives are important in predicting user satisfaction and continuance intentions.

Ryan et al. (2006) applied self-determination theory (SDT) in investigating the influence of psychological need satisfaction for Massively Multiplayer Online (MMO) computer game play. They found that SDT-derived measures of autonomy, competence, and relatedness need satisfactions independently predicted intentions for future game play in MMO setting.

In summary, these studies provide evidence that several factors, both hedonic and utilitarian, affect the acceptance of digital games. This was corroborated by Hamari et al. (2015), in a review of studies on adoption and use of digital games. It seems that the most typical way to measure acceptance has been via behavioral intention to use/play or continue using/playing. From the pool of most typically measured antecedents, attitude, flow, satisfaction, perceived enjoyment, and perceived playfulness were found to be the strongest predictors for use. The review also shows the severe lack of studies focusing on VR gaming acceptance as none were identified in this 2015 published review.

### Virtual reality gaming and related acceptance research

Various studies have been conducted on VR use in general. The perspective of the studies has ranged from more technology-centric studies to more user-centric studies. As examples of technology-centric VR studies, Ropelato et al. (2021) and Thomas and Rosenberg (2019) presented and examined algorithms for “hyper-reoriented walking” and “redirected walking” in VR, that is, steering algorithms that continuously redirect the user in order to make the virtual environment larger than the available physical space. As an example of a more user-centric VR study, Toyoda et al. (2021), among others, investigated the drivers of immersive virtual reality adoption intention. Most of the technology-centric VR studies have focused on VR systems in general with gaming as just one of the application areas, whereas numerous user-centric VR studies have focused on VR gaming particularly. In general, the majority of VR gaming studies seem to have a more user-centric approach.

On a general level, some user-centric VR gaming research has focused on hedonic or utilitarian perspective, but oftentimes separately. From the hedonic perspective, scholars have studied, for example, enjoyment (Frommel et al. 2017; Lin et al. 2018; Shafer et al. 2019; Sweetser and Rogalewicz 2020), immersion and presence (e.g., Lemmens et al. 2022; Navarro et al. 2019; Pallavicini and Pepe 2019; Tan et al. 2015; Winkler et al. 2020), flow (e.g., Bian et al. 2016; Bodzin et al. 2021; Michailidis et al. 2019; Pallavicini and Pepe 2019), negative emotional outcomes (Lavoie et al. 2021), and general player experience (e.g., Huang 2019; Marre et al. 2021; Tan et al. 2015; Xu et al. 2020) in VR games. Research has shown that VR games, in comparison with a desktop alternative, can provide the players with a higher degree of flow, a deeper immersion, a richer engagement with passive game elements (i.e., objects that players cannot directly interact with), and enhanced game experiences (e.g., Pallavicini and Pepe 2019; Tan et al. 2015).

From the utilitarian perspective, scholars have studied, for example, the effects of VR gaming on balance (e.g., Rendon et al. 2012), its effects on pain reduction during medical procedures (e.g., Wong et al. 2020), its use for different kinds of rehabilitation and therapy (e.g., Aulisio et al. 2020; Borstad et al. 2018), its use in education and learning (e.g., Oyelere et al. 2020), its potential in conducting breathing exercises (Patibanda et al. 2017) and supporting mental well-being (e.g., Pallavicini and Pepe 2020), as well as physical exertion of VR gaming (e.g., Gomez et al. 2018; Perrin et al. 2019). VR games often require some sort of physical movement from the player and, thus, provide some level of physical exertion to their users. These kinds of VR games are forms of exergames (cf. Kari and Makkonen 2014; Mueller et al. 2016). Due to this exergaming character, many VR games also pose possibilities to promote physical activity and well-being and, thus, be considered as a potential way to tackle the problems of sedentary lifestyle, which are becoming increasingly prevalent in our society. Furthermore, previous research (e.g., Berkovsky et al. 2010) has suggested that physical activity and digital gaming can be combined without adverse effects on the overall experience and enjoyment of playing. Undeniably, popular VR exergames such as Beat Saber (Beat Games 2021) and Soundboxing (Maxint LLC 2021) have been acclaimed for their ability to provide physical activity and exercise (e.g., VR Fitness Insider 2017). Overall, it seems obvious that VR games can very well be played also for utilitarian reasons.

VR systems also have limitations. There are several inconveniences to be overcome by the hardware and content developers. These issues are typically not directly related to VR gaming but VR use in general. For example, the use of VR might cause cybersickness (e.g., disorientation, nausea, or similar symptoms) to some users (Kim et al. 2018; Tian et al. 2022). Furthermore, wearing a VR headset typically prevents the user from seeing the physical world, and hence, the user can accidentally bump into furniture, walls, or even other people (Kotaku 2016). Wearing VR equipment can also cause physical disturbances and discomfort (Gregory 2017; Penumudi et al. 2020; Yan et al. 2018). From a more technical standpoint, bad quality graphics, bugs, and complex controls can trigger negative user experiences (Farič et al. 2019). These inconvenience factors are seldom addressed in VR gaming acceptance research.

While VR gaming has been studied from different perspectives, the number of studies on the factors behind VR gaming acceptance and use is scarce. We are aware of only few studies (Jang and Park 2019; Kosa et al. 2020; Tsai et al. 2021) that have investigated the acceptance of VR games. Jang and Park (2019) found presence, enjoyment, and perceived cost to be direct predictors of acceptance (intention to use) with enjoyment and presence having a higher impact than perceived cost. They also found perceived control, interactivity, and display quality to be indirect predictors of acceptance. Tsai et al. (2021) found that immersion experience and attitude toward using VR games are direct predictors of use intention, and that perceived usefulness, perceived ease of use, and perceived playfulness are indirect predictors of use intention via their significant impact on attitude. Kosa et al. (2020) found that acceptance of VR games was predicted by perceived autonomy, competence, and focused concentration of the players.

While the studies concerning VR gaming have investigated utilitarian and inconvenience factors, the related acceptance research has mostly overlooked these factors and focused on their hedonic use. As we have presented, VR games can also be used for utilitarian purposes and include inconvenience factors. Therefore, there is a general gap in VR gaming acceptance research, which we address in the present study by taking the approach of VR games affording both hedonic and utilitarian benefits to their users and including inconvenience factors. As such, our study aims to provide a greater understanding of VR gaming acceptance and use.

## Theoretical model

In this section, we draw from the previous related literature to craft a model for acceptance and use of VR games, as well as describe the hypotheses in the theoretical model. We also further demonstrate VR games’ hedonic and dual-purposed use value, as well as the inconvenience factors.

### Background of our theoretical model

Our theoretical model for explaining the acceptance and use of VR games is based on the hedonic-motivation system acceptance model (HMSAM) (Lowry et al. 2013), which we extend by (1) adding utilitarian factors and (2) inconvenience factors pertinent to VR systems.

**Fig. 1:**
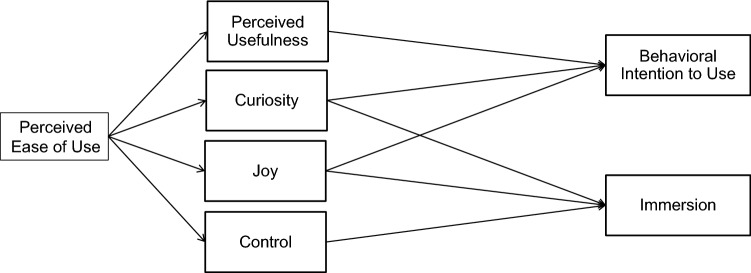
HMSAM (Lowry et al. 2013)

HMSAM is a hedonic-motivation system-specific acceptance model based on a theoretical perspective of flow-based cognitive absorption. The HMSAM draws and extends the van der Heijden’s (2004) model of hedonic system adoption by including cognitive absorption as a key mediator of perceived ease of use and of behavioral intentions to use hedonic-motivation systems. Figure 1 depicts the HMSAM. We use HMSAM as the backbone of our theoretical model representing the hedonic nature of VR gaming. HMSAM posits that curiosity and enjoyment predict intention to use and immersion. Perceived (hedonic) usefulness predicts intention to use, and control predicts immersion. Furthermore, HMSAM posits that perceived ease of use is an indirect predictor of intention to use and immersion, fully mediated by perceived (hedonic) usefulness, curiosity, enjoyment, and control.

Our first extension to HMSAM is the consideration of VR games as dual-purposed systems and adding the utilitarian factors to the model. More precisely, in addition to the hedonic factors, we examine the utilitarian factors of physical health and well-being (e.g., Aulisio et al. 2020; Borstad et al. 2018; Gomez et al. 2018; Perrin et al. 2019; Rendon et al. 2012; VR Fitness Insider 2017; Wong et al. 2020) and mental health and well-being (e.g., Pallavicini and Pepe 2020; Pallavicini et al. 2021). We posit that these are relevant for VR gaming and may potentially influence the acceptance and use of VR games.

Our second extension to HMSAM is adding the inconvenience factors to the model. More precisely, we examine the inconvenience factors of VR sickness (e.g., Kim, et al. 2018) and VR (physical) discomfort (e.g., Gregory 2017; Penumudi et al. 2020; Yan et al. 2018). We posit that these are relevant for VR gaming and may potentially influence the acceptance and use of VR games.

### Hypotheses

So as not to reproduce the hypotheses of the HMSAM (shown in Fig. 1), we present them only in a summarized form and refer the interested reader to ^1^van der Heijden (2004) and ^2^Lowry et al. (2013) for more details on these hypotheses. Following ^1^van der Heijden (2004) for H1a, H1c, H1e, H1g, and following ^2^Lowry et al. (2013) for H1b, H1d, H1f, H1h, H1i, H1j, our initial hypotheses emerge as follows:

- H1a: An increase in perceived ease of use will increase perceived usefulness.^1^
- H1b: An increase in perceived ease of use will increase curiosity.^2^
- H1c: An increase in perceived ease of use will increase enjoyment.^1^
- H1d: An increase in perceived ease of use will increase control.^2^
- H1e: An increase in perceived usefulness will increase intention to use.^1^
- H1f: An increase in curiosity will increase intention to use.^2^
- H1g: An increase in enjoyment will increase intention to use.^1^
- H1h: An increase in curiosity will increase immersion.^2^
- H1i: An increase in enjoyment will increase immersion.^2^
- H1j: An increase in control will increase immersion.^2^

#### Utilitarian-related hypotheses

Games can often be played for both hedonic and utilitarian purposes. Among other scholars, Arjoranta et al. (2020) showed that people play augmented reality games for both hedonic and utilitarian purposes and perceive both hedonic and utilitarian benefits from playing. Such utilitarian benefits include increased physical and mental well-being (Arjoranta et al. 2020). Others have presented the potential of VR games for physical (e.g., Gomez et al. 2018) and mental health and well-being (e.g., Pallavicini and Pepe 2020). Hedonic aspects of physical activity (e.g., Barkley and Penko 2009) and exergaming (e.g., Peng et al. 2011) have been shown to predict the amount of physical activity that an individual will conduct. In other words, increase in perceived (hedonic) usefulness has a positive influence on physical activity, and subsequently, this increased physical activity is likely to increase physical health and well-being perceptions. Further, Mohr et al. (2013) pointed out that a gameplay setting can enhance the benefits that people receive from therapeutic mental health interventions. Hence, we posit that perceived (hedonic) usefulness (as contextualized in HMSAM) could increase physical and mental health and well-being perceptions.

We further posit that physical health and well-being perceptions, as well mental health and well-being perceptions, could increase intention to use. While there seem to be no studies investigating these relationships directly within VR gaming, other gaming-related research imply for these associations. For example, Lin et al. (2012) found perceived exercise utility to have a significant impact on users' intention to play exergames. It was also shown that perceptions of performing exercise and having a better mood after a gaming session encourage the use of games (Kosa and Uysal 2020; Osorio et al. 2012). Arjoranta et al. (2020) showed that some people play an augmented reality game because it can increase their physical activity levels and support their mental well-being, that is, physical as well as mental health and well-being perceptions act as drivers for using a game.

Thus, we hypothesize

- H2a: An increase in perceived usefulness will increase physical health and well-being perceptions.
- H2b: An increase in perceived usefulness will increase mental health and well-being perceptions.
- H2c: An increase in physical health and well-being perceptions will increase intention to use.
- H2d: An increase in mental health and well-being perceptions will increase intention to use.

#### Inconvenience-related hypotheses

Compared to desktop gaming settings where players look at a computer or TV screen from a distance, VR headsets can be a bit more intrusive and uncomfortable for some people since they have their own weight and require physical contact with face (Cobb et al. 1999; Hu et al. 2011). Therefore, it is natural to expect some level of physical discomfort during VR experiences (Gregory 2017; Penumudi et al. 2020; Yan et al. 2018). If this experience is too overwhelming, this might consequently lead to a decrease in immersion levels and a decrease in the desire to use these kinds of systems.

Locomotion type is an important design decision in VR experiences. There are several ways of implementing movement in VR, including physical and artificial movement types (Boletsis and Cedergren 2019). Physical movement corresponds to user physically moving in real world with their headsets on, whereas artificial movement is implemented either via continuous movement (e.g., via continuous button press while staying still) or teleportation (e.g., via a button click while staying still). It has been found that ease of use of a controller can address the problem of discomfort, at least in teleportation cases (Boletsis and Cedergren 2019). Moreover, regardless of the locomotion style, poorly designed content, that is difficult to use, has been reported to be resulting in discomfort in VR users (Nichols 1999; Penumudi 2020).

Additionally, the immersiveness of a VR system sometimes comes with a cost because the visual sensation of the locomotion does not always perfectly match with the expectation of the user’s vestibular system. If the mismatch between what is seen and what is experienced is substantial, then a sensory conflict occurs and the feelings of simulation (VR) sickness follow (Shafer et al. 2019). For instance, in a roller coaster VR application, although the user is visually “told” that they are moving (i.e., accelerating and decelerating), their body does not feel that expected movement (i.e., in the case of continuous artificial movement). This makes users dizzy and disoriented (Boletsis and Cedergren 2019). Consequently, these feelings break the users’ sense of presence (immersion). At the same time, since VR sickness is a negative embodied experience (i.e., felt “in the body”), feeling of physical discomfort is a likely outcome for people who are experiencing VR sickness. Similarly, research shows that VR sickness also negatively influences the intention to adopt and use VR technology (Garrido et al. 2022; Sagnier et al. 2020).

Lastly, the experiences of physical discomfort and VR sickness can hinder players to have a positive user experience (e.g., Wang and Suh 2019) and it is possible that they also specifically hinder the perceived physical health and well-being benefits as they can cause the player to quit the gaming session altogether (Saredakis et al. 2020) making the session (and possible related physical activity) shorter. Further, it is plausible that an action (e.g., VR gaming) causing discomfort or sickness is likely to be associated with negative health and well-being perceptions. Therefore, we posit that the feelings of discomfort and VR sickness would be negatively associated to physical health and well-being perceptions.

Taken together, we hypothesize

- H3a: An increase in discomfort will decrease intention to use.
- H3b: An increase in VR sickness will decrease intention to use.
- H3c: An increase in discomfort will decrease immersion.
- H3d: An increase in VR sickness will decrease immersion.
- H3e: An increase in VR sickness will increase discomfort.
- H3f: An increase in perceived ease of use will decrease discomfort.
- H3g: An increase in discomfort will decrease physical health and well-being perceptions.
- H3h: An increase in VR sickness will decrease physical health and well-being perceptions.

### Proposed theoretical model

**Fig. 2:**
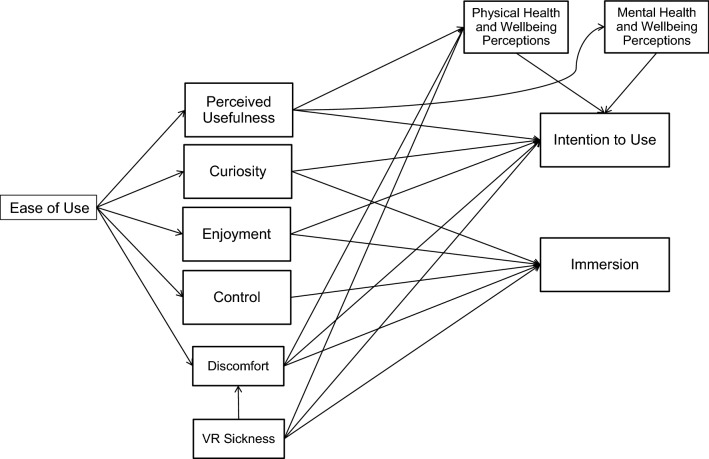
Proposed model for VR gaming acceptance and use

Figure 2 presents the proposed theoretical model. As the context of the present study is VR gaming, the proposed model is hence intended to be context specific. This approach allows to gain a deeper understanding of acceptance and user behavior within a specific context, which is considered valuable for both theory and practice (Burton-Jones and Straub 2006; Venkatesh et al. 2012).

## Methodology

To answer our research aims and assess the theorized relationships, we chose to use a quantitative confirmatory approach and conducted an online survey with VR gamers.

### Data collection

We selected online survey as the main data collection method because of its effectiveness in gathering large amounts of quantitative data. Online survey is a typical choice for data collection in these types of studies. The online survey was created with the LimeSurvey 3.15.8 software. Before launching the survey, the questionnaire was pre-tested qualitatively with five game and information systems scholars to ensure that the questions effectively captured the investigated topic. Based on the feedback, few minor modifications were made. The final questionnaire consisted of several sections, one of which was used to collect the data for testing the theoretical model. Other sections focused on demographics and on the usage habits of VR games. The final survey questionnaire was online for about two months during September 2020–October 2020. During this period, the online survey was actively promoted by distributing the survey invitation and link via social media (e.g., Facebook, Twitter, Reddit) and few VR and digital gaming-related discussion forums. The survey was targeted for people who had experience of playing virtual reality games and could thus give responses based on actual usage. For our analysis, we set a requirement that the latest gaming session had to be within the past six months. Before answering the survey, all the respondents gave their consent to use their responses in scientific research and in the researchers' publications. Answering the survey was anonymous. We also informed the respondents that the data would be analyzed with full anonymity and used for research purposes only. The average survey response time was about 11 min.

The descriptive questions on the demographics and usage habits were closed-ended multiple-choice questions, certain questions having an additional “Other, please specify” field if a suitable answer option was not listed (Tables 1 and 2 give a good indication of what was asked in terms of the demographics and usage habits). Regarding the questions for testing the theoretical model, we asked the respondents to rate statements concerning the use of VR games by using the traditional seven-point Likert scale consisting of response options ranging from strong disagreement to strong agreement. To avoid forced responses, the respondents also had the option “No response” with these questions. The statements (i.e., the wordings of the indicators) are presented in appendix Table 5. The questionnaire is available from the authors upon request.

**Table 1:** Demographics of the respondents (*N* = 473)

		(%)
Age		
≤ 25	213	45.0
26–35	130	27.5
36–45	82	17.3
≥ 46	48	10.2
Gender		
Male	418	88.3
Female	39	8.3
Other	8	1.7
Prefer not to say	8	1.7
Education		
No schooling completed	11	2.3
Primary education	41	8.7
Trade/technical/vocational education or equivalent	20	4.2
Further trade/technical/vocational education or equivalent	23	4.9
Upper secondary school, high school, gymnasium or equivalent	116	24.5
University of applied sciences degree or equivalent	42	8.9
Bachelor's degree (in college or university) or equivalent	130	27.5
Master's degree or equivalent	51	10.8
Doctorate or equivalent	17	3.6
No response	22	4.6
Country (≥ 10 respondents)		
Australia	16	3.4
Finland	17	3.6
Turkey	11	2.3
Germany	26	5.5
Sweden	13	2.7
United States	222	46.9
United Kingdom	42	8.9
Canada	29	6.1
Other	97	20.5

**Table 2:** Respondents’ VR gaming habits (*N* = 473)

		(%)
VR devices owned		
Standalone—6 degrees of freedom (6DoF)	95	20.1
Standalone—3 degrees of freedom (3DoF)	6	1.3
Tethered—6 degrees of freedom (6DoF)	335	70.8
Tethered—3 degrees of freedom (3DoF)	2	0.4
Mobile	7	1.5
Multiple devices	11	2.3
Other or cannot say	10	2.1
None, but has owned previously	4	0.8
None, has never owned any	3	0.6
VR play frequency		
I have only tried once or twice	3	0.6
Less than monthly	25	5.3
At least monthly	89	18.8
At least weekly	241	51.0
Daily	114	24.1
Cannot say	1	0.2
Main reasons for VR play		
Mainly for fun	314	66.4
Mainly for other reasons	3	0.6
Both fun and other reasons equally	156	33.0
Context of VR play		
Mainly alone	310	65.5
Mainly together with others in the same physical space	26	5.5
Mainly together with others over the network (online)	123	26.0
Other	14	3.0
Physical exertion level during VR play		
Mainly at LIGHT level (no accelerated breathing; no or only a very minor sensation of increased heart rate)	137	29
Mainly at MODERATE level (breathing quickens, but not out of breath; sensation of increased heart rate)	284	60
Mainly at VIGOROUS level (breathing is deep and rapid; strong sensation of increased heart rate)	49	10.4
Cannot say	3	0.6
Main location for VR play		
Mainly at home	469	99.2
Mainly at virtual reality arcade or similar	2	0.4
Mainly at office or workspace	1	0.2
Other	1	0.2

The focus of the present study was on VR games that are played using a VR headset as those are the most common and accessible options for the users. To ensure that the respondents provided answers based on VR games played with VR headsets, we instructed them as follows: “With virtual reality games, we refer to digital games that are played using a virtual reality headset. This activity can take place at home, in a virtual reality arcade, or in any other location.”

We conducted a power analysis prior to the data collection using pwrSEM (Wang and Rhemtulla 2021). The results showed that we needed at least 380 respondents for a 0.85 power. This was the power to detect a minimum effect size of 0.15 for the immersion-control relationship. The power of all other relationships were higher than 0.85.

### Measures

The 11 constructs in the theoretical model were operationalized to be measured by three to eight indicators based on existing scales from prior literature. Only minor modifications were made to the scales—we mostly adjusted the wording to fit the context of VR gaming. The measurement models of all the constructs were reflective.

The Perceived ease of use (PEOU), Perceived usefulness (PU), Curiosity (CUR), Enjoyment (ENJ), Control (CONT), Immersion (IMM), and Intention to use (BIU) constructs were adapted from Lowry et al. (2013). The Physical health and well-being perceptions (PHWB), Mental health and well-being perceptions (MHWB), and Discomfort (DIS) constructs were adapted from Kari and Makkonen (2014). The VR sickness (VRS) construct was adapted and modified (i.e., simplified) from Kim et al. (2018).

### Study respondents

A total of 506 VR gamers participated in the study. Thirty-three of the respondents stated that they haven’t played a VR game in the last 6 months and were thus discarded from the analysis. Therefore, the analyses were carried out with *N* = 473 respondents (Male = 418, Female = 39, Other = 8, Prefer not to say = 8). The average age of the respondents was 29.09 (SD = 10.96). More than half of the respondents had either high school (*n* = 116) or bachelor’s degree (*n* = 130). Almost half of the respondents resided in the USA (*n* = 222). Next most represented three countries were UK (n = 42), Canada (*n* = 29), and Germany (*n* = 26). The demographics of the respondents are summarized in Table 1 and further information on their VR gaming habits are presented in Table 2.

Regarding the respondents’ VR gaming habits, almost all respondents (*n* = 466) owned VR devices themselves. Significant proportion (*n* = 335) either owned “Tethered—6 degrees of freedom (6DoF) devices (e.g., Oculus Rift S, PlayStation VR, HTC VIVE Cosmos, Windows Mixed Reality)” or “Standalone—6 degrees of freedom (6DoF) devices (e.g., Oculus Quest, HTC VIVE Focus, Lenovo Mirage Solo)” (*n* = 95). Most of the respondents stated that they play VR games at least weekly (*n* = 355 of which *n* = 114 were playing daily). Also, 314 of the respondents stated that they mainly play VR games for fun, whereas three were playing them mainly for other reasons, and 156 for both fun and other reasons equally. Moreover, 310 respondents stated that they mainly play VR games alone, 123 played mainly together with others over the network (online), whereas 26 stated they mainly play co-located with others. Most of the players were playing VR games mainly at least at moderate level (*n* = 333 of which *n* = 49 stated they play mainly at vigorous level). Finally, almost all of the respondents mainly played VR games in home setting (*n* = 469).

## Analysis and results

We analyzed our theoretical model through covariance-based SEM using IBM SPSS and AMOS Statistics 22.0. Before reporting the model estimation results, we discuss the reliability and validity of its constructs and their indicators, the potential common method bias, and the overall goodness of fit of the estimated model with the data.

### Indicator and construct reliability and validity

The reliability and validity of the model indicators were analyzed using standardized confirmatory factor analysis (CFA) loadings. The indicator loadings (lambda values) are expected to be statistically significant and greater than or equal to 0.50 (Hair et al. 2010). The loadings of four indicators were lower than 0.50 (i.e., VRS1, PEOU2, IMM4, and IMM5). After discarding those four indicators, all indicators were loading adequately in their respective constructs. Indicator loadings are presented in appendix Table 5.

All skewness and kurtosis values for discomfort, VR Sickness, mental health and well-being perceptions, and physical health and well-being perceptions were between 1.0 and −1.0, which implied that there was no normality violation in our data.

Next, we ran a confirmatory factor analysis (CFA). It is recommended that the Comparative Fit Index (CFI) ≥ 0.90, Tucker–Lewis Index (TLI) ≥ 0.90, root-mean-square error of approximation (RMSEA) ≤ 0.06, and standardized root mean square residual (SRMR) ≤ 0.08 (Hu and Bentler 1999; Gefen et al. 2011). The results showed that the model fit was good (CFI = 0.94, TLI = 0.93, RMSEA = 0.043, SRMR = 0.05).

For construct validities, we examined the convergent and discriminant validity of the constructs.

For convergent validity, each construct should have an average variance extracted (AVE) greater than or equal to 0.50. That is, on average, each construct should explain at least half of the variance of the construct’s indicators (Fornell and Larcker 1981). In our case, AVE values were found to be exceeding 0.50, except for perceived usefulness, control, discomfort, virtual reality sickness, and immersion. However, the composite reliabilities of these constructs were above 0.70, which made them acceptable in terms of ensuring convergent validity (Fornell and Larcker 1981; Hair et al. 2010). The AVE of each construct is reported in the last row of Table 3.

**Table 3:** Correlation matrix: CRs, AVEs, square roots of AVEs (on-diagonal cells; bolded), and correlations (off-diagonal cells) of the constructs

	PEOU	PU	CUR	ENJ	CONT	DIS	VRS	PHWB	MHWB	BIU	IMM
PEOU	**.77**										
PU	.52**	**.69**									
CUR	.44**	.55**	**.79**								
ENJ	.61**	.52**	.56**	**.85**							
CONT	.62**	.45**	.46**	.60**	**.67**						
DIS	−.44**	−.35**	−.26**	−.41**	−.39**	**.66**					
VRS	−.29**	−.13*	−.10*	−.23**	−.18**	.50**	**.63**				
PHWB	.23**	.51**	.33**	.23**	.25**	−.20**	.001	**.88**			
MHWB	.30**	.64**	.34**	.31**	.26**	−.18**	−.003	.62**	**.89**		
BIU	.51**	.43**	.55**	.73**	.53**	−.32**	−.16^**^	.21^**^	.22**	**.93**	
IMM	.46**	.48**	.50**	.53**	.44**	−.19**	.08	.26^**^	.28**	.50**	**.69**
CR	.79	.78	.84	.85	.83	.69	.75	.92	.92	.96	.78
AVE	.60	.47	.63	.73	.45	.43	.40	.78	.80	.88	.48

For discriminant validity, each construct should have a square root of AVE greater than or equal to its absolute correlation with the other constructs. That is, on average, each construct should share at least an equal proportion of variance with its indicators as it shares with the other constructs (Fornell and Larcker 1981). All constructs met this criterion. The square root of AVE of each construct (on-diagonal cells) and the correlations between the constructs and their statistical significance (off-diagonal cells) are reported in Table 3.

For construct reliabilities, we examined the composite reliabilities (CR) of the constructs. All of them, except for discomfort, were above 0.70 suggesting good reliability (Fornell and Larcker 1981; Nunnally and Bernstein 1994). The CR of discomfort was 0.69, which may still be considered acceptable (Fornell and Larcker 1981), and we chose to include it. The CR of each construct is reported in the second last row of Table 3.

### Common method bias tests

We also tested for common method bias. Harman’s single factor accounted for a variance of 29.8%, which is acceptable for the commonly accepted threshold of 50% (Podsakoff et al. 2003; as used in Pavlou et al. 2007). Additionally, unmeasured latent factor method showed that the common variance was 22%. Moreover, all variance inflation factors (VIF) were less than 3.3, which is an indication of no common method bias (Kock 2015). Taken together, we concluded that there is no indication of common method bias in our data.

### Model estimation

As for our main analysis, we ran a structural equation model (SEM). We assessed the goodness of fit of the estimated model using four alternative fit indices recommended in the literature (Hu and Bentler 1999): CFI, TLI, RMSEA, and SRMR. The results showed that all four fit indices (CFI = 0.90, TLI = 0.90, RMSEA = 0.052, SRMR = 0.07) suggested an acceptable fit by meeting the respective cutoff criteria (CFI ≥ 0.90, TLI ≥ 0.90, RMSEA ≤ 0.06, and SRMR ≤ 0.08) suggested by Hu and Bentler (1999), as well as Gefen et al. (2011).

**Fig. 3:**
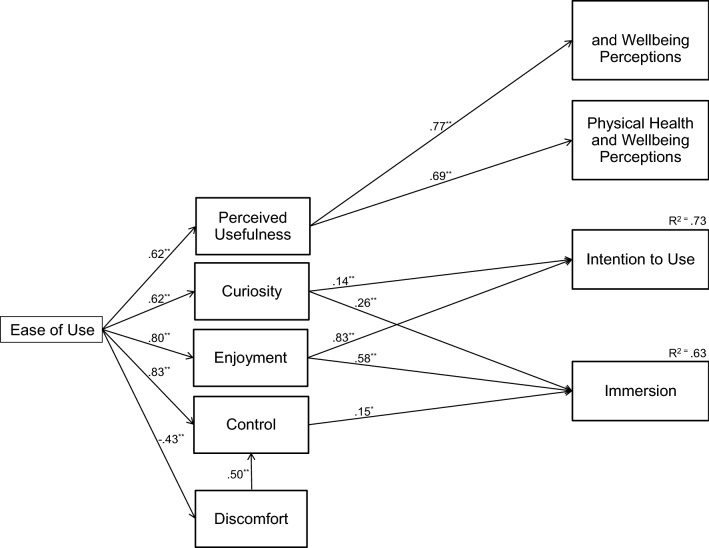
The structural model (significant paths only)

The model estimation results in terms of the standardized size of the statistically significant effects, as well as the proportion of explained variance (R^2^), are reported in Fig. 3. Figure 3 also depicts our final proposed model with significant paths only.

As can be seen, from the hypothesized effects, H1a, H1b, H1c, H1d, H1e, H2b, H2c, H3a, H3b, H3c, H4a, H4b, and H4f were statistically significant, whereas no support was found for H2a, H2d, H2e, H2f, H2g, H3d, H3e, H4c, and H4d. More precisely, perceived ease of use was associated positively with perceived usefulness, curiosity, enjoyment, and control, whereas it was negatively associated with discomfort. Perceived usefulness predicted physical and mental well-being perceptions; VR sickness predicted discomfort; and curiosity, enjoyment, and control predicted immersion. Behavioral intention to use was predicted by curiosity and enjoyment. As can be seen, some of the significant associations, as shown in the correlation matrix (Table 3), did not persist in the path model (e.g., PU -> BIU). These are summarized in Table 4. The explained variances (R^2^) of immersion and intention to use were 0.63 and 0.73, respectively.

**Table 4:** Hypotheses and whether they are supported by the correlation matrix or path model

Hypothesis	Support from correlation matrix	Support from path model
H1a: PEOU-> PU	Yes	Yes
H1b: PEOU-> CUR	Yes	Yes
H1c: PEOU-> ENJ	Yes	Yes
H1d: PEOU-> CONT	Yes	Yes
H1e: PU-> BIU	Yes	No
H1f: CUR-> BIU	Yes	Yes
H1g: ENJ-> BIU	Yes	Yes
H1h: CUR-> IMM	Yes	Yes
H1i: ENJ-> IMM	Yes	Yes
H1j: CONT-> IMM	Yes	Yes
H2a: PU-> PHWB	Yes	Yes
H2b: PU-> MHWB	Yes	Yes
H2c: PHWB-> BIU	Yes	No
H2d: MHWB-> BIU	Yes	No
H3a: DIS-> BIU	Yes	No
H3b: VRS-> BIU	Yes	No
H3c: DIS-> IMM	Yes	No
H3d: VRS-> IMM	No	No
H3e: VRS-> DIS	Yes	Yes
H3f: PEOU-> DIS	Yes	Yes
H3g: DIS-> PHWB	Yes	No
H3h: VRS-> PHWB	No	No

## Discussion

The present study aimed to explain the factors that drive the use and acceptance of VR gaming along with VR gaming immersion. We built on the HMSAM by Lowry et al. (2013), which we extended by adding utilitarian factors and inconvenience factors to the model in order to capture the pertinent features of VR systems.

The performance of the model in terms of the proportion of explained variance was found to be very good, as it was able to explain 73% of the variance in the intention to use VR games and 63% of the variance in the immersion. This was in spite of the fact that only two out of the seven constructs that were hypothesized to affect intention to use were found to have a statistically significant effect on it, whereas three out of the five constructs that were hypothesized to affect immersion were found to have a statistically significant effect on it. The constructs affecting intentions to use were curiosity and enjoyment, and the constructs affecting immersion were curiosity, enjoyment, and control. In addition, we found that perceived ease of use was associated positively with perceived usefulness, curiosity, enjoyment, and control, whereas it was negatively associated with discomfort. Perceived usefulness predicted physical and mental well-being perceptions, and VR sickness predicted discomfort.

From a purely confirmatory perspective, the relatively small number of hypothesized effects (13/22) that were statistically significant can perhaps be considered somewhat disappointing. However, from an exploratory perspective, an examination of not only which of the effects were found as statistically significant, but also which of them were found as statistically insignificant, as well as the differences in the effect sizes, provide several interesting theoretical and practical implications. As such, the study provides a twofold contribution. First, we provide a greater theoretical understanding on VR gaming acceptance and use. Second, our study contributes to the more general research stream on VR systems’ acceptance.

### Contributions to research

The primary contribution of the present study concerns theorizing and validating a model for understanding VR gaming acceptance, whereas prior research has seen VR games mainly as hedonic systems and focused on their leisure use, we addressed the concept more holistically also including the utilitarian use aspects and inconvenience factors in the theorized model. Our model provides evidence on VR gaming acceptance and helps explain the use of VR games, as well as other outcomes. At the same time, our findings contribute to the understanding of general VR systems’ acceptance and use.

One of the central findings concerns the significant effects of enjoyment and curiosity on the intention to use VR games in comparison with the insignificant effects of utilitarian and inconvenience factors, which suggests that VR gaming is driven more by the hedonic gaming aspects than by the utilitarian health and well-being aspects of these games. In fact, although utilitarian and inconvenience factors were associated with VR gaming intentions without the influence of other variables (Table 3), contrary to our hypotheses, they seem to play no role behind VR gaming intentions according to the results from our path model (Fig. 3, Table 4). This means that people play VR games mainly because they are fun, not because they would promote one’s health and well-being. As VR games appear to be prominently seen as hedonic systems, this also, in a way, explains the insignificant effect of perceived usefulness to VR gaming intentions—even if conceptualized as perceived usefulness for hedonic purposes. While the path was significant in the HMSAM (Lowry et al. 2013), several other studies have shown the lack of relevance (e.g., Hsu and Lu 2004) in digital gaming context. However, perceived usefulness had a significant role behind the utilitarian factors of mental health and well-being perceptions and physical health and well-being perceptions, indicating that perceived (hedonic) usefulness may also increase utilitarian perceptions of VR gaming. Further, the reason behind utilitarian factors’ insignificant effect on use intention is not in that VR games would not provide physical exertion, as 60% of the respondents reported that their physical exertion level during VR play is mainly moderate and for 10%, it was mainly vigorous. While this provides further evidence to the discussion on VR games’ exergaming nature, it also shows that utilitarian health and well-being aspects do not seem to act as drivers for use intention. This is mostly in line with previous findings on non-VR exergames (e.g., Kari and Makkonen 2014; Lin et al. 2012), which have shown that for use intention, the effect of perceived enjoyment is greater than the effect of perceived utility. These findings are, of course, about user perceptions. The users might be benefiting from the gaming activities without consciously realizing it.

As another important finding, we found inconvenience factors to not affect the VR gaming intention and immersion. This can be seen as surprising considering prior research, which has shown that VR sickness negatively influences intention to use VR systems (e.g., Sagnier et al. 2020). This leads us to suspect that either the VR sickness and discomfort have become decreasingly common issues in novel VR system use or that the strong effect of enjoyment overrules these negative sensations. However, it should also be noted that these relationships were significant as zero-order correlations (except for VR sickness and immersion), which then disappear when all variables are included in the path model (Table 4). Also contrary to our hypotheses, neither discomfort nor VR sickness was associated with physical health and well-being perceptions. This indicates that the players experiencing these issues consider them irrelevant of the physical health and well-being perceptions and likely see them as quickly passing issues that leave no negative outcomes after being over, whereas the players perceive the outcomes related to physical health and well-being as more longer-term outcomes that are not affected by temporary inconveniences. Perceiving VR sickness and discomfort as quickly passing issues might also partly explain why they do not affect the VR gaming intention. Relating to this, as hypothesized, we did find VR sickness to affect discomfort. VR sickness being a negative embodied experience (i.e., felt “in the body”) also results in feelings of increased physical discomfort.

Another interesting finding was that control was not found to be associated with intention to use, contrary to research stating that autonomy is one of the influential factors for gaming motivations (Ryan et al. 2006). This might be due to the relatively small amount of VR games available in the market in comparison with the non-VR alternatives, where respondents might have been implicitly thinking that they generally have more control in desktop or console games that are offering more freedom compared to their experience of existing VR games possibly having limited features, and thus reported lower on the control statements. The fully immersive nature of VR or the curiosity toward novel experiences might also be decreasing the importance of control. Future research of habitual players of acclaimed VR games might shed more light into the control-intention to use relationship.

As hypothesized, immersion was affected by curiosity, enjoyment, and control, whereas no support was found for the other hypothesized paths (i.e., inconvenience factors) concerning immersion. It should be noted that, while significant, the effects of curiosity on both the intention to use and immersion were rather weak, same as the effect of control on immersion. Respectively, enjoyment acts as the strongest predictor of immersion. This means that for an immersive VR gaming experience to take place, it is more important to enjoy the game play than the experience of curiosity or the feeling of control. Enjoyment being the strongest predictor is also in line with the literature examining enjoyment as a sub-component or a prerequisite for immersion (e.g., Jennett et al. 2008). Also, Lowry et al. (2013), referring to Guo and Poole (2009) and Webster et al. (1993), state that “Flow theory posits that if a person enjoys his or her interactions and has intrinsic motivation to perform some task, immersion or flow is a logical causal outcome.” However, it is worth noting that the actual causal direction of the enjoyment-immersion relationship is not clear given the cross-sectional nature of the data.

Ease of use, as hypothesized, had a direct positive effect on perceived usefulness, curiosity, enjoyment, and control, as well as a direct negative effect on discomfort. Further, it had an indirect effect on the intention to use and immersion mediated by curiosity, enjoyment, and control, as well as an indirect effect on mental and physical health and well-being perceptions mediated by perceived usefulness. These associations are not surprising, as the important role of ease of use in digital gaming context has been shown in several studies before (e.g., Hsu and Lu 2004; Kosa et al. 2020; Tsai et al. 2021) to the extent that it can be seen as a basic requirement for digital games (Sweetser and Wyeth 2005). A completely new finding is the indirect effect of ease of use on mental and physical health and well-being perceptions mediated by perceived (hedonic) usefulness. This finding further emphasizes the importance of ease of use in digital gaming context, as it is shown to be important not just for hedonic purposes but also for utilitarian purposes. Further, easy to use VR games might also alleviate physical discomfort occurring due to headset use.

As the HMSAM (Lowry et al. 2013) was used as the backbone for our theoretical model, we reflect our results in the light of the HMSAM. Similar to HMSAM, immersion was predicted by enjoyment, curiosity, and control. Also, as proposed by the HMSAM, enjoyment and curiosity predicted intention to use, but in the present study, perceived usefulness did not. This was the sole difference between the propositions of the HMSAM and our significant paths.

In addition to the findings derived from testing the theoretical model, our study also reveals some general gaming habits of VR gamers. Of the VR gamers who responded our survey, about 75% play VR games at least weekly, and of them, 24% daily. This suggests that those playing VR games are relatively active gamers in general. The gaming mainly takes place in home setting, whereas VR arcades or other locations are rarely the main setting for gaming—not meaning that people would not play in these setting, but that playing at home is the primary choice. This is to be expected considering that almost all respondents also owned VR devices themselves. Playing alone is about twice as popular as playing together with others. Interestingly, about two thirds were playing VR games mainly for fun and a third mainly for both fun and other reasons equally. This suggests that some people have intentions to play VR games for utilitarian purposes alongside hedonic purposes, however, we were not able to spot this in our model. It might be that our health and well-being focused utilitarian factors were not comprehensive enough to capture the full range of utilitarian factors. That is, apart from their hedonic motivations, players might have other utilitarian purposes than health and well-being in mind when playing. Another explanation could be that hedonic motivations simply overrule the utilitarian health and well-being motivations in terms of intention to use. This would seem as a plausible explanation as more than two thirds were mainly playing VR games at moderate-to-vigorous exertion level. Lastly, by far the most owned devices are ones with 6DoF, of which tethered are more commonly owned than standalone.

### Contributions to practice

The findings of the present study can be utilized by several stakeholders. As our main practical contribution, we provide implications for the providers of VR games by suggesting different actions and aspects that are valuable to consider in the design and development process of these games.

Entertainment and utility form two interesting aspects of VR gaming and link VR gaming to dual-purposed use. Considering the strong effect of enjoyment behind the intention to use, fun and enjoyment aspects should be the spearhead in designing and developing VR games. Same applies for the marketers of VR games. Even for the marketers of VR exergames, focusing on the enjoyment aspects would seem to be a more favorable choice than focus on utilitarian health and well-being aspects. Following this, about 70% of the respondents reported that their physical exertion level during VR play is mainly moderate or vigorous, while at the same time, utilitarian factors were not associated with intention to use. This shows that while people play VR games mainly for fun, they still receive exercise benefits. This means that the utilitarian exercise benefits are a side-product of the gaming. Thus, VR games could be a potential tool to increase the physical activity levels of those who are otherwise more interested on gaming than physical activity. Additionally, if VR games would somehow present the physical health and well-being benefits of playing to the user, general perceptions of the utilitarian benefit could be stronger. This might also lead to the increased popularity of VR gaming, if more people would perceive it as a way of making physical activity more attractive, beyond the enjoyment of playing the games.

Considering the important role of ease of use, VR games should be designed to provide frictionless interfaces, logical and easy to use controls, and easy setup in daily use. A well-designed onboarding would aid the players in learning how to operate the games and also VR systems in general, as the controls are perhaps not that standardized yet as with other forms of digital games. Ease of use can also lessen the experiencing of discomfort while playing.

We further suggest the designers and developers of VR games to produce games utilizing the 6DoF of VR devices, as those devices were the most typically owned ones and can also more likely produce more enjoyable gaming experiences. 6DoF devices in comparison with 3DoF devices can also induce more physical movement for the players. As playing in most cases takes place in home settings, the providers of VR games would likely benefit from focusing primarily on such VR games that can be played with typical VR devices in home setting, and only secondarily on games that are played in VR arcades. Of course, some games can be played equally in both. This would likely also result positively in the general popularity of VR systems in home use. For those mainly playing VR games in a multiplayer setting, playing together with others over the network (i.e., online) is much more popular than playing in the same location. Considering that the owners of VR devices quite seldom own multiple similar devices, this is expected. However, it suggests that if a VR game provider would be producing a game with multiplayer features, they should primarily focus on such multiplayer modes that can be played online.

Steffen et al. (2019) argue that many practitioners lack a comprehensive understanding of the drivers of VR adoption. We expect that the stakeholders in the VR gaming industry will benefit from our findings and implications by gaining a more complete understanding of how to develop VR games that are perceived as both fun and useful by the players and achieve commercial success, subsequently also increasing the general popularity and diffusion of VR systems.

### Limitations and future research

This study has certain limitations. First, we acknowledge that other antecedents besides those investigated in this study might be associated with VR gaming intention and immersion. Thus, we encourage future research to extend on our findings and investigate other factors possibly related to VR gaming. Particularly, future studies might consider other utilitarian factors that might play a role in VR gaming acceptance. Second, this study used self-reported cross-sectional survey data to produce an understanding of VR gaming. It could be beneficial to use also other methods (such as interviews) to investigate the factors associated with VR gaming. Qualitative studies directly asking players about their gaming reasons in an open-ended fashion might reveal valuable information about their intentions. Further, longitudinal studies could be conducted to examine if and how the drivers behind use intentions change during the course of time. For example, it would be an interesting research avenue to investigate and compare the significant drivers at the early and later stages of use. Third, even though our survey was meant for all demographics of VR gamers and distributed via channels where we expected to get a varying reach, our sample turned out to be very male oriented. This could be due to VR gamer segment being male dominant or due to us not being able to reach the non-male VR gamers. This naturally poses some restrictions to generalizability. Also, the conclusions that can be drawn from this study in terms of the adoption and diffusion of VR technology are somewhat limited by the composition of the sample (this being participants who already had experience of playing virtual reality games). However, we particularly focused on this segment as we wanted the participants to be able to give responses based on actual usage. Yet, it is plausible that, at this point in the diffusion cycle, our sample has consisted of people who are, for example, relatively unsusceptible to VR sickness and the results concerning inconvenience factors might reflect more the experiences of those who are predisposed toward greater comfort with immersive media. Fourth, our study focused on VR gaming with headsets in general. Since there are different settings to play VR games, for example homes and VR arcades, future research is needed to focus on these particular settings or compare the results between different settings. Fifth, as the data collection took place during the COVID-19 pandemic, we cannot rule out the potential confounding impact that restrictions on movement and public gatherings in many countries may have had on motivations for using VR technology during the data collection period. Lastly, we needed to discard some items from our scales to ensure the validity of our measurement model. Future research can take these into account and try alternative scales when studying VR gaming acceptance.

Additional avenues for future research include comparing the performance of the presented context-specific model to more general models commonly used to explain the acceptance and use of technology. It would also be interesting to see how the presented model would perform in the case of VR systems in general (i.e., non-game context). Considering our finding that the inconvenience factors did not affect intention, future research could tap into this relationship in more depth by using qualitative methods. Furthermore, expectations of users might play a role in multi-motive information systems use as well (Lowry et al. 2015). Therefore, expectation and confirmation variables can be included in future models to see whether they have an effect on the use intentions or whether they suppress other relationships. Future research should also aim to replicate our study with more female oriented sample to increase the generalizability of the findings. Another potential avenue for future research would be to investigate how the produced insights and understanding could be applied in the design and development process of VR games.

## Conclusion

In this paper, we investigated the factors that drive the use and acceptance of VR games. We first proposed a theoretical model based on the HMSAM, which we extended by adding utilitarian and inconvenience factors to capture the pertinent features of VR systems. We then analyzed the model through SEM using an online survey sample of 473 VR gamers. In summary, our findings help explain the role of different antecedents behind VR gaming acceptance. The results of the study revealed that (1) VR gaming is driven more by the hedonic gaming aspects than by the utilitarian health and well-being aspects of VR games, (2) use intentions and immersion levels are not significantly diminished by physical discomfort and VR sickness, and (3) ease of use plays an important role in VR gaming context, as it was shown to be important not just for hedonic purposes but also for utilitarian purposes. The study provides a twofold contribution. First, we provide a greater theoretical understanding on VR gaming acceptance and use. Second, our study contributes to the more general research stream on VR systems’ acceptance. With our findings and by suggesting various actions that would be valuable to consider in the design and development process of VR games, we help VR game designers and developers to provide such VR gaming solutions that people genuinely want to use and invest in. Based on our study, we also pinpoint potential avenues for future research.

## Appendix

See Table

**Table 5:** Measurement scales and indicator loadings

Construct	Indicators	Indicator loadings	Based on
Enjoyment	Joy1. I find playing VR games to be enjoyable	.88	Adapted from Lowry et al. (2013) to a VR gaming context
	Joy2. I have fun using VR games	.87	
	Joy3. Using VR games is boring*	.75	
	Joy4. VR games really annoy me*	.52	
	Joy5. VR gaming experience is pleasurable	.77	
	Joy6. VR games leave me unsatisfied*	.53	
Control	CTL1. I have a lot of control when playing VR games	.74	Adapted from Lowry et al. (2013) to a VR gaming context
	CTL2. I can choose freely what I want to see or do when playing VR games	.68	
	CTL3. I have little control over what I can do when playing VR games*	.62	
	CTL4. I am in control when playing VR games	.77	
	CTL5. I have no control over my interaction when playing VR games*	.52	
	CTL6. I am allowed to control my interaction when playing VR games	.67	
Immersion	IMM1. I am able to block out most other distractions when playing VR games	.63	Adapted from Lowry et al. (2013) to a VR gaming context
	IMM2. I am absorbed in what I am doing when playing VR games	.84	
	IMM3. I am immersed in the game when playing VR games	.77	
	IMM4. I am distracted by other attentions very easily when playing VR games*	< 0.50 discarded	
	IMM5. My attention is not diverted very easily when playing VR games	< 0.50 discarded	
Curiosity	CUR1. VR gaming experience excites my curiosity	.87	Adapted from Lowry et al. (2013) to a VR gaming context
	CUR2. VR gaming experience makes me curious	.82	
	CUR3. VR gaming experience arouses my imagination	.68	
Perceived ease of use	PEOU1. My interaction with VR games is clear and understandable	.73	Adapted from Lowry et al. (2013) to a VR gaming context
	PEOU2. Interacting with VR games does not require a lot of my mental effort	< 0.50 discarded	
	PEOU3. I find VR games to be trouble free	.51	
	PEOU4. I find it easy to get VR games to do what I want them to do	.66	
	PEOU5. Learning to operate VR games is easy for me	.70	
	PEOU6. It is simple to do what I want with VR games	.71	
	PEOU7. It is easy for me to become skillful at using VR games	.50	
	PEOU8. I find VR games easy to use	.70	
Perceived usefulness	PU1. VR games decrease my stress	.70	Adapted from Lowry et al. (2013) to a VR gaming context
	PU2. VR games help me better pass time	.62	
	PU3. VR games provide a useful escape	.60	
	PU4. VR games help me think more clearly	.62	
	PU5. VR games help me feel rejuvenated	.59	
Discomfort	DIS1. Playing virtual reality games feels to me as physically disturbing	.61	Adapted from Kari and Makkonen (2014) to a VR gaming context
	DIS2.Playing virtual reality games feels to me as physically uncomfortable	.71	
	DIS3. Playing virtual reality games feels to me as physically inconvenient	.63	
Physical health and well-being perceptions	PHWB1. Playing virtual reality games helps me to better maintain my physical health	.89	Adapted from Kari and Makkonen (2014) to a VR gaming context
	PHWB2. Playing virtual reality games helps me to better maintain my physical ability to function	.84	
	PHWB3. Playing virtual reality games helps me to better maintain my physical well-being	.92	
Mental health and well-being perceptions	MHWB1. Playing virtual reality games helps me to better maintain my mental health	.94	Adapted from the (P)HWB construct in Kari and Makkonen (2014) to a VR gaming context
	MHWB2. Playing virtual reality games helps me to better maintain my mental ability to function	.82	
	MHWB3. Playing virtual reality games helps me to better maintain my mental well-being	.91	
Virtual reality sickness	VRS1. Playing virtual reality games occasionally causes me fatigue	< 0.50 discarded	Modified (i.e., simplified) original scale from Kim et al. (2018)
	VRS2. Playing virtual reality games occasionally causes me eyestrain	.57	
	VRS3. Playing virtual reality games occasionally causes me headache	.75	
	VRS4. Playing virtual reality games occasionally causes me dizziness	.64	
	VRS5. Playing virtual reality games occasionally causes me nausea	.68	
Behavioral intention to use	BIU1. I plan on using VR games in the future	.93	Adapted from Lowry et al. (2013) to a VR gaming context
	BIU2. I intend to continue using VR games in the future	.95	
	BIU3. I expect my use of VR games to continue in the future	.93	

*Reverse coded

5.

## Footnotes

1 Flow is defined as optimal experiences characterized by having a skill level just enough to overcome presented obstacles (Csikszentmihalyi 1990).

## References

[CR1] Agarwal R, Karahanna E (2000). Time flies when you're having fun: cognitive absorption and beliefs about information technology usage. MIS Q.

[CR2] Angelov V, Petkov E, Shipkovenski G, Kalushkov T (2020) Modern virtual reality headsets. In: 2020 International congress on human-computer interaction, optimization and robotic applications, pp 1–5. IEEE (2020)

[CR3] Arjoranta J, Kari T, Salo M (2020). Exploring features of the pervasive game Pokémon GO that enable behavior change: qualitative study. JMIR Serious Games.

[CR4] Aulisio MC, Han DY, Glueck AC (2020). Virtual reality gaming as a neurorehabilitation tool for brain injuries in adults: a systematic review. Brain Inj.

[CR5] Barkley JE, Penko A (2009). Physiologic responses, perceived exertion, and hedonics of playing a physical interactive video game relative to a sedentary alternative and treadmill walking in adults. J Exerc Physiol.

[CR6] Beat Games (2021) Beat Saber. https://beatsaber.com. Accessed 28 Oct 2021

[CR7] Berkovsky S, Coombe M, Freyne J, Bhandari D, Baghaei N (2010) Physical activity motivating games: virtual rewards for real activity. In: Proceedings of the SIGCHI conference on human factors in computing systems, pp 243–252. ACM

[CR8] Bian Y, Yang C, Gao F, Li H, Zhou S, Li H, Meng X (2016) A framework for physiological indicators of flow in VR games: construction and preliminary evaluation. Pers Ubiquit Comput 20:821–832

[CR9] Bodzin A, Junior RA, Hammond T, Anastasio D (2021). Investigating engagement and flow with a placed-based immersive virtual reality game. J Sci Educ Technol.

[CR10] Boletsis C, Cedergren JE (2019). VR locomotion in the new era of virtual reality: an empirical comparison of prevalent techniques. Adv Hum-Comput Interact.

[CR11] Borstad AL, Crawfis R, Phillips K, Lowes LP, Maung D, McPherson R, Gauthier LV (2018). In-home delivery of constraint-induced movement therapy via virtual reality gaming. J Patient-Centered Res Rev.

[CR12] Burdea GC, Coiffet P (1994). Virtual reality technology.

[CR13] Burton-Jones A, Straub DW (2006). Reconceptualizing system usage: an approach and empirical test. Inf Syst Res.

[CR14] Chang IC, Liu CC, Chen K (2014). The effects of hedonic/utilitarian expectations and social influence on continuance intention to play online games. Internet Res.

[CR15] Cobb SV, Nichols S, Ramsey A, Wilson JR (1999) Virtual reality-induced symptoms and effects (VRISE). Presence: Teleoperators Virtual Environ 8:169–186

[CR16] Cruz-Neira C, Sandin DJ, DeFanti TA (1993) Surround-screen projection-based virtual reality: the design and implementation of the CAVE. In: Proceedings of the 20th annual conference on computer graphics and interactive techniques, pp 135–142. ACM

[CR17] Cummings JJ, Bailenson JN (2016). How immersive is enough? A meta-analysis of the effect of immersive technology on user presence. Media Psychol.

[CR18] Csikszentmihalyi M (1990). Flow: the psychology of optimal experience.

[CR19] Farič N, Potts HW, Hon A, Smith L, Newby K, Steptoe A, Fisher A (2019). What players of virtual reality exercise games want: thematic analysis of web-based reviews. J Med Internet Res.

[CR20] Flavián C, Ibáñez-Sánchez S, Orús C (2019). The impact of virtual, augmented and mixed reality technologies on the customer experience. J Bus Res.

[CR21] Forbes (2018) VR Wave Breaking Outside the Home. https://www.forbes.com/sites/charliefink/2018/05/28/vr-wave-breaking-outside-the-home/#67259296770e. Accessed 1 Dec 2021

[CR22] Fornell C, Larcker DF (1981). Evaluating structural equation models with unobservable variables and measurement error. J Mark Res.

[CR104] Frommel J, Sonntag S, Weber M (2017) Effects of controller-based locomotion on player experience in a virtual reality exploration game. In: 12th international conference on the foundations of digital games. ACM, pp 1–6

[CR23] Garrido LE, Frías-Hiciano M, Moreno-Jiménez M, Cruz GN, García-Batista ZE, Guerra-Peña K, Medrano LA (2022) Focusing on cybersickness: pervasiveness, latent trajectories, susceptibility, and effects on the virtual reality experience. Virtual Reality, 1–25 10.1007/s10055-022-00636-4 PMC8886867 35250349

[CR24] Gefen D, Rigdon EE, Straub D (2011). Editor’ s comments: an update and extension to SEM guidelines for administrative and social science research. MIS Q.

[CR25] Gomez DH, Bagley JR, Bolter N, Kern M, Lee CM (2018). Metabolic cost and exercise intensity during active virtual reality gaming. Games Health J.

[CR26] Grand View Research (2020a) Virtual Reality in Gaming Market Size, Share & Trends Analysis Report by Component, By Device, By User (Commercial Space, Individual), By Region, And Segment Forecasts, 2020–2027. https://www.grandviewresearch.com/industry-analysis/virtual-reality-in-gaming-market. Accessed 1 Dec 2021

[CR27] Grand View Research (2020b) Virtual Reality Headset Market Size, Share & Trends Analysis Report by End-device (Low-end, High-end), By Product Type (Standalone, Smartphone-enabled), By Application (Gaming, Education), And Segments Forecasts, 2021–2028. https://www.grandviewresearch.com/industry-analysis/virtual-reality-vr-headset-market. Accessed 1 Dec 2021

[CR28] Gregory J (2017). Virtual reality.

[CR29] Guo YM, Poole MS (2009). Antecedents of flow in online shopping: a test of alternative models. Inf Syst J.

[CR30] Hair JF, Black WC, Babin BJ, Anderson RE (2010). Multivariate data analysis.

[CR31] Hamari J, Keronen L, Alha K (2015) Why do people play games? A review of studies on adoption and use. In: 2015 48th Hawaii International conference on system sciences, pp 3559–3568, IEEE

[CR32] Hong SJ, Tam KY (2006). Understanding the adoption of multipurpose information appliances: the case of mobile data services. Inf Syst Res.

[CR33] Hsu CL, Lu HP (2004). Why do people play on-line games? an extended TAM with social influences and flow experience. Inf Manag.

[CR34] Hu LT, Bentler P (1999). Cutoff criteria for fit indexes in covariance structure analysis: conventional criteria versus new alternatives, structural equation modeling. Struct Equ Model.

[CR35] Hu B, Ma L, Zhang W, Salvendy G, Chablat D, Bennis F (2011). Can virtual reality predict body part discomfort and performance of people in realistic world for assembling tasks?. Int J Ind Ergon.

[CR36] Huang W (2019) Exploring players' user experience in a high-embodied virtual reality game. In: 2019 IEEE games, entertainment, media conference, pp 1–8. IEEE

[CR37] Jabil (2018) The future of augmented and virtual reality gaming: taking the tech mainstream. https://www.jabil.com/blog/gaming-will-drive-augmented-and-virtual-reality-adoption.html. Accessed 1 Dec 2021

[CR38] Jang Y, Park E (2019). An adoption model for virtual reality games: the roles of presence and enjoyment. Telematics Inform.

[CR39] Jennett C, Cox AL, Cairns P, Dhoparee S, Epps A, Tijs T, Walton A (2008). Measuring and defining the experience of immersion in games. Int J Hum Comput Stud.

[CR40] Kari T (2019) Virtual reality arcades: a study on usage habits with emphasis on digital gaming. In: International conference on videogame sciences and arts. Springer, Cham, pp 179–194

[CR41] Kari T, Makkonen M (2014) Explaining the usage intentions of exergames. In: Proceedings of the 35th international conference on information systems. AIS, pp 1–18

[CR42] Kim HK, Park J, Choi Y, Choe M (2018). Virtual reality sickness questionnaire (VRSQ): motion sickness measurement index in a virtual reality environment. Appl Ergon.

[CR43] Kim S, Yun JH (2020). Motion-aware interplay between WiGig and WiFi for wireless virtual reality. Sensors.

[CR44] Kock N (2015). Common method bias in PLS-SEM: a full collinearity assessment approach. Int J e-Collab.

[CR45] Kosa M, Uysal A (2020) Four pillars of healthy escapism in games: Emotion regulation, mood management, coping, and recovery. In: Game user experience and player-centered design. Springer, Cham, pp 63–76

[CR46] Kosa M, Uysal A, Eren PE (2020). Acceptance of virtual reality games: a multi-theory approach. Int J Gaming Comput-Mediated Simul.

[CR47] Kotaku (2016) Seven stories of injuries and other VR hazards. https://kotaku.com/seven-stories-of-injuries-and-other-vr-hazards-1756697518. Accessed 25 Oct 2021

[CR48] Lavoie R, Main K, King C, King D (2021). Virtual experience, real consequences: the potential negative emotional consequences of virtual reality gameplay. Virtual Reality.

[CR49] Lemmens JS, Simon M, Sumter SR (2022). Fear and loathing in VR: the emotional and physiological effects of immersive games. Virtual Reality.

[CR50] Lin HH, Wang YS, Chou CH (2012). Hedonic and utilitarian motivations for physical game systems use behavior. Int J Hum-Comput Interact.

[CR51] Lin JHT, Wu DY, Tao CC (2018). So scary, yet so fun: the role of self-efficacy in enjoyment of a virtual reality horror game. New Media Soc.

[CR52] Lowry PB, Gaskin J, Moody GD (2015). Proposing the multi-motive information systems continuance model (MISC) to better explain end-user system evaluations and continuance intentions. J Assoc Inf Syst.

[CR53] Lowry PB, Gaskin J, Twyman N, Hammer B, Roberts T (2013). Taking ‘fun and games’ seriously: proposing the hedonic-motivation system adoption model (HMSAM). J Assoc Inf Syst.

[CR54] Marre Q, Caroux L, Sakdavong JC (2021). Video game interfaces and diegesis: the impact on experts and novices’ performance and experience in virtual reality. Int J Hum-Comput Interact.

[CR55] Maxint LLC (2021) Soundboxing. https://www.soundboxing.co. Accessed 1 Dec 2021

[CR56] Michailidis L, Barcias JL, Charles F, He X, Balaguer-Ballester E (2019) Combining personality and physiology to investigate the flow experience in virtual reality games. In: International conference on human–computer interaction. Springer, Cham, pp 45–52

[CR57] Mohr DC, Burns MN, Schueller SM, Clarke G, Klinkman M (2013). Behavioral intervention technologies: evidence review and recommendations for future research in mental health. Gen Hosp Psychiatry.

[CR58] Mueller F, Khot RA, Gerling K, Mandryk R (2016) Exertion games. Foundations and Trends® in Human–Computer Interaction 10, 1–86

[CR59] Muhanna MA (2015). Virtual reality and the CAVE: taxonomy, interaction challenges and research directions. J King Saud Univ-Comput Inf Sci.

[CR60] Mostafa AE, Ryu WHA, Chan S, Takashima K, Kopp G, Costa Sousa M, Sharlin E (2017) Designing NeuroSimVR: a stereoscopic virtual reality spine surgery simulator. University of Calgary Science Research and Publications, pp 1–20

[CR61] Navarro R, Vega V, Martinez S, Espinosa MJ, Hidalgo D, Benavente B (2019) Designing experiences: a virtual reality video game to enhance immersion. In: International conference on applied human factors and ergonomics. Springer, Cham, pp 233–242

[CR62] Nichols S (1999). Physical ergonomics of virtual environment use. Appl Ergon.

[CR63] Nunnally JC, Bernstein IH (1994). Psychometric theory.

[CR64] Osorio G, Moffat DC, Sykes J (2012). Exergaming, exercise, and gaming: sharing motivations. Games Health J.

[CR65] Oyelere SS, Bouali N, Kaliisa R, Obaido G, Yunusa AA, Jimoh ER (2020). Exploring the trends of educational virtual reality games: a systematic review of empirical studies. Smart Learn Environ.

[CR66] Pallavicini F, Orena E, di Santo S, Greci L, Caragnano C, Ranieri P, Mantovani F (2021) MIND-VR: design and evaluation protocol of a virtual reality psychoeducational experience on stress and anxiety for the psychological support of healthcare workers involved in the covid-19 pandemic. Front Virtual Real 2:620225

[CR67] Pallavicini F, Pepe A (2019) Comparing player experience in video games played in virtual reality or on desktop displays: immersion, flow, and positive emotions. In: Extended abstracts of the annual symposium on computer–human interaction in play companion extended abstracts. ACM, pp 195–210

[CR68] Pallavicini F, Pepe A (2020). Virtual reality games and the role of body involvement in enhancing positive emotions and decreasing anxiety: within-subjects pilot study. JMIR Serious Games.

[CR69] Patibanda R, Mueller FF, Leskovsek M, Duckworth J (2017) Life tree: understanding the design of breathing exercise games. In: Proceedings of the annual symposium on computer–human interaction in play. ACM, pp 19–31

[CR70] Pavlou PA, Liang H, Xue Y (2007). Understanding and mitigating uncertainty in online exchange relationships: a principal-agent perspective. MIS Q.

[CR71] Peng W, Lin JH, Crouse J (2011). Is playing exergames really exercising? A meta-analysis of energy expenditure in active video games. Cyberpsychol Behav Soc Netw.

[CR72] Penumudi SA, Kuppam VA, Kim JH, Hwang J (2020). The effects of target location on musculoskeletal load, task performance, and subjective discomfort during virtual reality interactions. Appl Ergon.

[CR73] Perrin T, Faure C, Nay K, Cattozzo G, Sorel A, Kulpa R, Kerhervé HA (2019). Virtual reality gaming elevates heart rate but not energy expenditure compared to conventional exercise in adult males. Int J Environ Res Public Health.

[CR74] Podsakoff PM, MacKenzie SB, Lee JY, Podsakoff NP (2003). Common method biases in behavioral research: a critical review of the literature and recommended remedies. J Appl Psychol.

[CR75] Rendon AA, Lohman EB, Thorpe D, Johnson EG, Medina E, Bradley B (2012). The effect of virtual reality gaming on dynamic balance in older adults. Age Ageing.

[CR76] Ropelato S, Menozzi M, Huang MYY (2021) Hyper-reoriented walking in minimal space. Virtual Reality, pp 1–9

[CR77] Ryan RM, Rigby CS, Przybylski A (2006). The motivational pull of video games: a self-determination theory approach. Motiv Emot.

[CR78] Sagnier C, Loup-Escande E, Lourdeaux D, Thouvenin I, Valléry G (2020). User acceptance of virtual reality: an extended technology acceptance model. Int J Hum–comput Interact.

[CR106] Saredakis D, Szpak A, Birckhead B, Keage HA, Rizzo A, Loetscher T (2020) Factors associated with virtual reality sickness in head-mounted displays: a systematic review and meta-analysis. Front Hum Neurosci 14:96 10.3389/fnhum.2020.00096 PMC7145389 32300295

[CR79] Shafer DM, Carbonara CP, Korpi MF (2019). Factors affecting enjoyment of virtual reality games: a comparison involving consumer-grade virtual reality technology. Games Health J.

[CR80] Sherman WR, Craig AB (2018). Understanding virtual reality: interface, application, and design.

[CR81] Statista (2016) Virtual reality (VR) video gaming sales revenue worldwide from 2016 to 2020, by segment. https://www.statista.com/statistics/538801/global-virtual-reality-gaming-sales-revenue-segment/. Accessed 1 Dec 2021

[CR82] Statista (2021) Virtual reality (VR) gaming revenue worldwide from 2017 to 2024. https://www.statista.com/statistics/499714/global-virtual-reality-gaming-sales-revenue/. Accessed 1 Dec 2021

[CR83] Steffen JH, Gaskin JE, Meservy TO, Jenkins JL, Wolman I (2019). Framework of affordances for virtual reality and augmented reality. J Manag Inf Syst.

[CR84] Sweetser P, Rogalewicz Z (2020) Affording enjoyment in VR games: possibilities, pitfalls, and perfection. In: 32nd Australian conference on human–computer interaction, pp 55–64. ACM

[CR85] Sweetser P, Wyeth P (2005). GameFlow: a model for evaluating player enjoyment in games. Comput Entertain.

[CR86] Tan CT, Leong TW, Shen S, Dubravs C, Si C (2015) Exploring gameplay experiences on the oculus rift. In: Proceedings of the 2015 annual symposium on computer–human interaction in play. ACM, pp 253–263.

[CR87] Techopedia (2017) Head-Mounted Display (HMD). https://www.techopedia.com/definition/2342/head-mounted-display-hmd. Accessed 2 Dec 2021

[CR88] Thomas J, Rosenberg ES (2019) A general reactive algorithm for redirected walking using artificial potential functions. In: Proceedings of the 2019 IEEE conference on virtual reality and 3D user interfaces. IEEE, pp 56–62

[CR89] Tian N, Lopes P, Boulic R (2022) A review of cybersickness in head-mounted displays: raising attention to individual susceptibility. Virtual Reality, pp 1–33

[CR90] Toyoda R, Russo Abegão F, Gill S, Glassey J (2021) Drivers of immersive virtual reality adoption intention: a multi-group analysis in chemical industry settings. Virtual reality, 1–12 10.1007/s10055-021-00586-3 PMC8494627 34642566

[CR91] Tsai JC, Chen LY, Peng HY (2021). Exploring the factors influencing consumer's attitude toward using and use intention of virtual reality games. Int J Organ Innov.

[CR105] Van der Heijden H (2004) User acceptance of hedonic information systems. MIS Q 28:695–704

[CR92] Venkatesh V, Thong JY, Xu X (2012). Consumer acceptance and use of information technology: extending the unified theory of acceptance and use of technology. MIS Q.

[CR93] Venturebeat (2018) VR arcades are playing a leading role in the consumer market. https://venturebeat.com/2018/07/05/vr-arcades-are-playing-a-leading-role-in-the-consumer-market. Accessed 28 Oct 2021

[CR94] Verge (2021) What is the metaverse, and do I have to care? One part definition, one part aspiration, one part hype. https://www.theverge.com/22701104/metaverse-explained-fortnite-roblox-facebook-horizon. Accessed 21 Dec 2021

[CR95] VR Fitness Insider (2017) Job Stauffer’s story about losing 60 pounds and reclaiming his health with VR. https://www.vrfitnessinsider.com/job-stauffers-story-losing-60-pounds-reclaiming-health-vr. Accessed 28 Oct 2021

[CR96] Wang G, Suh A (2019) User adaptation to cybersickness in virtual reality: a qualitative study. In: Proceedings of the 27th European conference on information systems. AIS, pp 1–15

[CR97] Wang YA, Rhemtulla M (2021). Power analysis for parameter estimation in structural equation modeling: a discussion and tutorial. Adv Methods Pract Psychol Sci.

[CR98] Webster J, Trevino LK, Ryan L (1993). The dimensionality and correlates of flow in human-computer interaction. Comput Hum Behav.

[CR99] Winkler N, Röthke K, Siegfried N, Benlian A (2020) Lose yourself in VR: exploring the effects of virtual reality on individuals’ immersion. In: Proceedings of the 53rd Hawaii international conference on system sciences. AIS, pp 1510–1519

[CR100] Wong J, Ghiasuddin A, Tamaye H, Siu A (2020). Effectiveness of virtual reality gaming on pain reduction in children during PIV/PICC placement. J Technol Behav Sci.

[CR101] Xi N, Hamari J (2021). Shopping in virtual reality: a literature review and future agenda. J Bus Res.

[CR102] Xu W, Liang HN, Zhang Z, Baghaei N (2020). Studying the effect of display type and viewing perspective on user experience in virtual reality exergames. Games Health J.

[CR103] Yan Y, Chen K, Xie Y, Song Y, Liu Y (2018) The effects of weight on comfort of virtual reality devices. In: International conference on applied human factors and ergonomics. Springer, Cham, pp 239–248

